# Fish assemblage structure, diversity and controls on reefs of South Kona, Hawaiʻi Island

**DOI:** 10.1371/journal.pone.0287790

**Published:** 2023-07-06

**Authors:** Atsuko Fukunaga, Gregory P. Asner, Bryant W. Grady, Nicholas R. Vaughn

**Affiliations:** Center for Global Discovery and Conservation Science, Arizona State University, Hilo, Hawaii, United States of America; PLOS ONE, UNITED KINGDOM

## Abstract

The structure of coral-reef fish assemblages is affected by natural and anthropogenic factors such as the architectural complexity, benthic composition and physical characteristics of the habitat, fishing pressure and land-based input. The coral-reef ecosystem of South Kona, Hawai‘i hosts diverse reef habitats with a relatively high live coral cover, but a limited number of studies have focused on the ecosystem or the fish assemblages. Here, we surveyed fish assemblages at 119 sites in South Kona in 2020 and 2021 and investigated the associations between the fish assemblages and environmental variables obtained from published Geographic Information System (GIS) layers, including depth, latitude, reef rugosity, housing density and benthic cover. The fish assemblages in South Kona were dominated by a relatively small number of widely occurring species. Multivariate analyses indicated that fish assemblage structure strongly correlated with depth, reefscape-level rugosity and sand cover individually, while the final parsimonious model included latitude, depth, housing density within 3-km of shore, chlorophyll-*a* concentration and sand cover. Univariate analysis revealed negative associations between housing density and fish species richness and abundance. Effects of environmental factors specific to fish trophic groups were also found. Reefscape-level rugosity had strong positive influences on the distributions of all herbivores (browsers, grazers and scrapers), while housing density had strong negative influences only on the abundance of browsers. Positive associations were also found between live coral cover and the presence of scrapers, as well as the abundance of corallivorous fish. This study intensively surveyed shallow coral reefs along the coastline of South Kona and was the most complete spatial survey on the reef fish assemblages to date. As it utilized GIS layers to assess large-scale patterns in the fish assemblages, future studies including in-situ environmental data may further reveal local-scale patterns and insights into factors affecting the structure of fish assemblages in Hawai‘i.

## Introduction

The structure of coral-reef fish assemblages, such as species composition and abundance, are affected by both physical and biological factors, including the architectural complexity of the reefs, depth and benthic composition [1–4]. The architectural complexity of coral reefs arising from underlying topography and coral morphologies generally has positive effects on fish abundance, but the effects can vary among different trophic groups and dependent on the spatial resolutions of the complexity (e.g., fine-scale vs. reefscape-level rugosity) [5]. Depth also differentially affects fish trophic groups, with, for example, herbivores being more abundant in shallower waters than deeper waters [6]. For benthic composition, high levels of live coral cover and coral species richness are generally linked to high fish abundance and species richness [7], while the abundances of algae and coral influences herbivorous and corallivorous fish distributions that prey on these organisms [4, 5].

Anthropogenic stressors can also affect the structure of reef fish assemblages. Increases in fishing pressure negatively impacts the distribution and species richness of reef fish [8], as well as biomass [9] and size structure by disproportionately affecting large fishes [10]. Land-based pollution, such as nitrogen input from sewage disposal systems, can also reduce fish biomass [9]. Increases in sedimentation affect the distribution of coral colonies and in turn the use of habitat by reef fish [11]. Marine heatwaves associated with climate change and resulting coral bleaching and mortality can reduce the abundance of reef fish, particularly corallivores, and the level of local human disturbance affects the speed of recovery [12]. It is, therefore, essential to consider these anthropogenic factors, as well as physical and biological factors, when examining the state of coral-reef fish assemblages.

**Fig 1:**
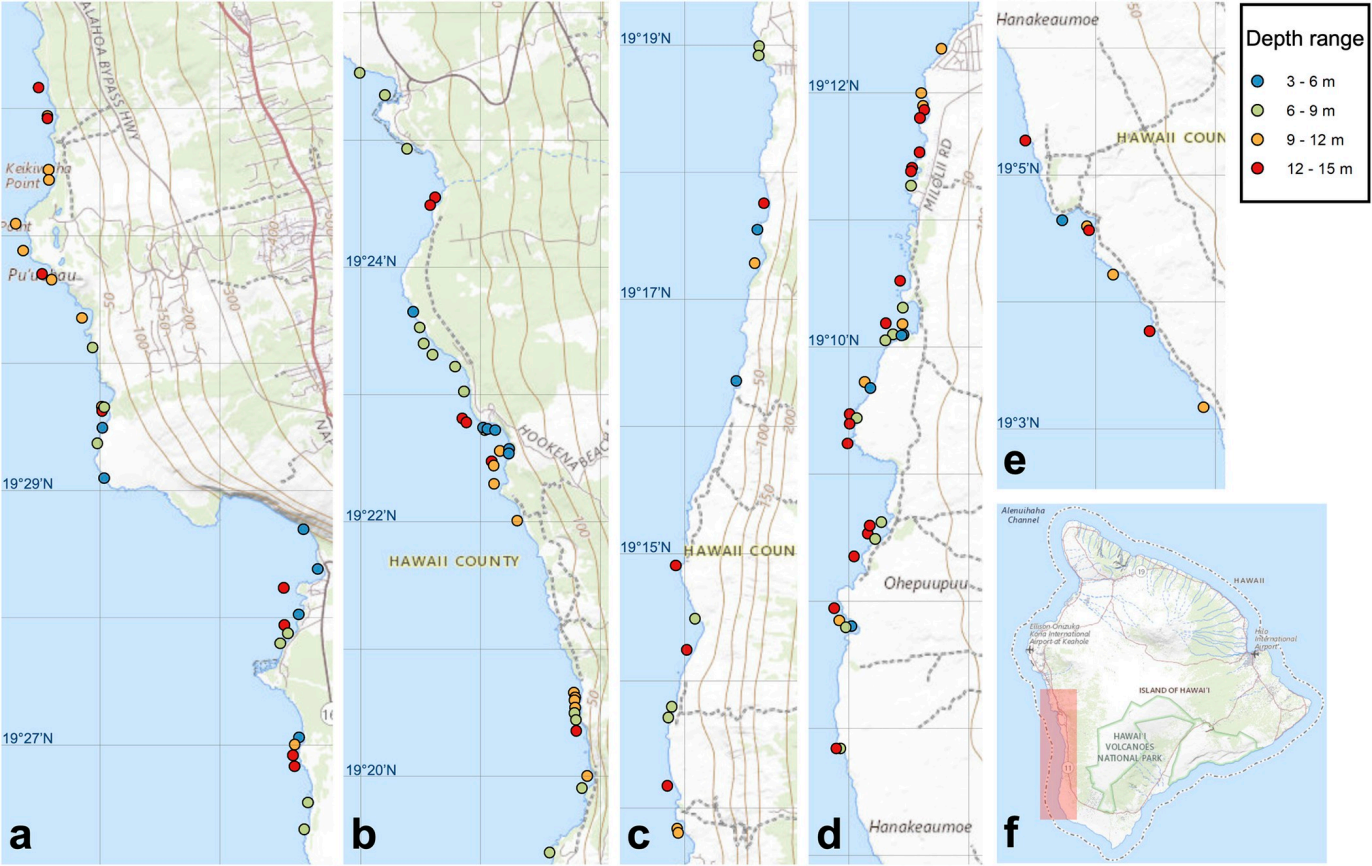
Maps of survey sites in South Kona from north to south ends (a-e) and Hawai‘i Island (f) showing South Kona. The survey sites are categorized by depth ranges and the area of surveys in South Kona is shaded in red. Background image: USGS The National Map.

South Kona District on the island of Hawai‘i extends along a 60.5-km coastline on the southwestern side of the island (Fig 1). Located south of Kailua-Kona where the highest human population density on the western side of Hawai‘i Island occurs, the human population density in South Kona is highest at the north end, decreasing toward the south end [13, 14]; there are a combination of residential housing and dense smallholder agricultural operations in the northern portion and less-dense human populations with fewer but larger agricultural operations in the southern portion. The amounts of impervious surface and associated surface water runoff, density of on-site sewage disposal systems and non-commercial fishing pressure also follow this overall pattern of gradient from north to south along the coastline [14].

The coral-reef ecosystem of South Kona hosts diverse reef habitats with a relatively high live coral cover [15] and varying habitat structural complexity and physical characteristics [13]. While the shallow-water coral reefs in South Kona generally have clear waters with visibility often exceeding 30 m due to lower levels of land-based input in comparison to other areas of Hawai‘i Island, the northern embayment of Hōnaunau receive an elevated level of sediment load from runoff [16]. Coral reefs towards the southern end of South Kona are also relatively inaccessible in comparison to those on the northern end mainly due to restricted or difficult road access to the shoreline, limiting their recreational use. These differences in coral-reef habitats and gradients in the population density and anthropogenic activities along the coast of South Kona provided an opportunity to investigate potential factors driving the distribution of reef fish in Hawai‘i.

Here we describe a study examining the fish assemblages from 119 shallow coral-reef sites along the coastline of South Kona. Despite its high live coral cover, a limited number of studies have focused on the coral reef ecosystem of South Kona, whereas there have been a relatively large number of reef studies and coordinated efforts along the northwest coast of Hawai‘i Island (e.g., [17–20]). We conducted intensive, repeated surveys of 119 sites in 2020 and 2021 along the 60.5-km coastline and then utilized published geographic information system (GIS) layers to investigate the patterns of the fish assemblages in each year in relation to large-scale environmental factors, such as depth, latitude, reef rugosity, benthic cover and housing density. This study serves as baseline data of fish assemblages in South Kona and provides insights into potential drivers of the structure of reef fish assemblages in Hawai‘i.

## Materials and methods

### Ethics statement

Permits were not required for our observational surveys in South Kona. Hawai‘i Division of Aquatic Resources Special Activity Permits apply only to removal and manipulations of benthic community.

### Fish surveys

Fish survey sites were selected along the coastline using a stratified random sampling design as described in Asner et al., 2021 [13]. Briefly, Random Forest Machine Learning analysis was used to identify environmental variables that were associated with live coral cover and reef rugosity at 2-m resolutions (reefscape-level rugosity), two variables known to correlate strongly with fish abundance (e.g., Friedlander et al., 2007 [21]). Based on this analysis, *k*-means clustering was done using latitude, depth, reef curvature, macroalgal cover, along with live coral cover and reefscape-level rugosity as input variables. The optimal number of strata, 18, was determined using Gap statistic [22] and an iterative sample-size reduction analysis. The number of survey sites within each of the 18 strata was then determined by the relative size of the stratum and the standard deviation of live coral cover and reefscape-level rugosity within the stratum.

A set of 119 sites (Fig 1) selected using the stratified random sampling design were surveyed twice, once each in 2020 and 2021 between June and August. Survey sites ranged from 3 m to 15 m in depth. At each site, a pair of divers laid two 25-m transect tapes in parallel, and each diver completed a 25 m × 5 m belt transect survey for diurnally active reef fish along the tapes while swimming at a constant rate approximately 10 m apart from one another. The time required to complete the survey was approximately 15 minutes per transect. All fishes were identified to the lowest possible taxonomic level (mostly species) and also categorized by trophic habits based on Heenan et al. (2016) [23] and unpublished data from Hawai‘i Division of Aquatic Resources. Resource fishes that are targeted reef fishery species in Hawai‘i were also determined based on unpublished data from Hawai‘i Division of Aquatic Resources. Abundance-occupancy relationship.

To investigate the abundance-occupancy relationship (i.e., relationship between species abundance and the number of sites they occupy [24]) of reef fish in South Kona, we calculated for each year numerical dominance of each species (the total count of each species from the 119 survey sites), occurrence of each species (the number of sites where each species occurred) and mean density of each species (mean count of each species per survey site when they occurred, ignoring the sites with 0 counts). To focus on resident reef fishes, pelagic and semi-pelagic fishes such as sharks and rays, jacks, barracudas and silversides were removed prior to the calculation of these three metrics. While numerical dominance and occurrence allowed for investigations into widely occurring, numerically abundant species, mean density could identify species that were not overall numerically dominant in South Kona but were abundant when they occurred.

### Fish assemblage patterns

Environmental variables (Table 1) for data analyses were obtained from published GIS layers and included depth and reefscape-level rugosity (2-m resolution) from the Global Airborne Observatory (GAO) [25], housing density (as tax parcel density) within 1-km radius and 3-km radius of the nearest point on shore (Hawai‘i State Tax Map), the number of unique vegetation types within 3-km radius of the nearest point on shore [26], and live coral cover, algal cover and sand cover at 2-m resolution from the GAO [15]. UTM northing coordinates (latitude) were also obtained based on GPS coordinates of survey sites. Average sea surface temperature, wave height and chlorophyll-*a* concentration data from the Pacific Islands Ocean Observing System’s Ocean Tipping Points [16] were also considered (https://www.pacioos.hawaii.edu/projects/oceantippingpoints/#data), but only chlorophyll-*a* concentration was retained as average sea surface temperature and wave height were highly correlated with UTM northing coordinates and chlorophyll-*a* concentration. The number of unique vegetation types within 1-km radius of the nearest point on shore was also considered but not included due to a high correlation with the number of unique vegetation types within 3-km radius (Table 1).

**Table 1:** List of environmental variables considered for inclusion in statistical analyses.

Variables	Source
UTM northing coordinates	GPS coordinates
Depth (2 m)	[25]
Rugosity (2 m)	
Housing density within 1-km radius of the nearest point on shore **	Hawai‘i State Tax Map
Housing density within 3-km radius of the nearest point on shore	
Number of unique vegetation types within 1-km radius of the nearest point on shore *	[26]
Number of unique vegetation types within 3-km radius of the nearest point on shore	
Live coral cover (2 m)	[15]
Algal cover (2 m)	
Sand cover (2 m)	
Chlorophyll-*a* concentration (4 km)	[16]
Average sea surface temperature (5 km) *	
Wave height (0.5 km) *	

GIS cell resolutions are shown in parentheses where applicable. The variables shown with “*” were not included in the analyses due to their high correlations with other environmental variables. Housing density within 1-km radius of the nearest point on shore (marked with “**”) was also excluded prior to multivariate DISTLM conditional test and univariate BRT analyses as this variable did not have statistically significant marginal correlation with the fish assemblage structure.

To investigate the relationships between environmental variables and fish assemblages, multivariate distance-based linear models (DISTLM) [27, 28] were constructed separately for 2020 and 2021 using the software package PRIMER 7 [29] with the PERMANOVA+ add-on [30]. The rationale for analyzing the 2020 and 2021 data separately was that, due to the use of GIS layers to obtain environmental variables, there were no distinct values of the environmental variables corresponding to each year, so a single model considering “year” as a factor could not be built. For each year, fish count data from two surveyors were combined for the number of individuals per specie per survey site (250 m^2^). Pelagic and semi-pelagic fishes were removed to focus on resident reef fishes. The structure of fish assemblages as a whole was calculated using the Bray-Curtis dissimilarity measure after square-root transformation of the number of individuals per species per site. DISTLM performs two different tests (marginal and conditional tests), and p-values for testing the null hypothesis of no relationship for explanatory variables are obtained using a permutation method. DISTLM marginal tests test for the relationship between a response variable and an individual explanatory variable alone, ignoring other explanatory variables, while conditional tests examine the amount of variation explained by an explanatory variable after one or more explanatory variables have been fitted, considering the overlap in the variation explained by multiple explanatory variables.

Due to the large number of environmental variables, we first performed DISTLM marginal tests to investigate the relationship between each environmental variable and the fish assemblage structure on the basis of the Bray-Curtis dissimilarity separately. Housing density within 1-km radius of the nearest point on shore was removed from DISTLM conditional tests, as well as univariate analyses (see below for details), since this variable did not explain a significant proportion of variations in the fish assemblage structure. For DISTLM conditional tests, appropriate parsimonious models for the 2020 and 2021 data were selected based on the small-sample-size corrected version of Akaike’s information criterion (AICc). All explanatory variables (i.e., environmental variables) were normalized prior to the analyses, and 4999 permutations of residuals under a reduced model [31] were used to obtain p-values for testing the null hypothesis for the explanatory variables. Note that we did not include strata that were used for our survey site selection as a factor because environmental variables used for the stratification were directly included as numerical explanatory variables in our analyses.

We next used Boosted Regression Trees (BRT) for univariate analyses to investigate the relationships between the environmental variables obtained from the GIS datasets and the abundances of reef fish at the 119 survey sites. Response variables used in the univariate analyses were the total fish abundance, species richness, abundances of fish trophic groups (grazers, browsers, scrapers and corallivores) and abundance of resource fish at each site. The same variables from the GIS datasets as in the multivariate DISTLM analysis were used as explanatory variables (i.e., northing, depth, reefscape-level rugosity, housing density within 3-km radius, unique vegetation types within 3-km radius, live coral cover, algal cover and sand cover). After preliminary analyses evaluating the potential probability distribution to model the response variables, abundance response variables (i.e., total abundance, abundance by trophic group and abundance of resource fish) were log transformed (log[y+1]) and the Gaussian probability distribution was chosen. For the abundance of scrapers, a further data transformation to presence/absence was necessary as the data were sparse, and the Bernoulli probability distribution was used for the analysis. Species richness was modeled using the Poisson probability distribution.

All BRT univariate analyses were completed in the statistical software R version 4.1.1 [32] with the gbm [33] and dismo [34] packages. The model for each response variable was fitted using the gbm.step function of the dismo package. The function assesses the optimal number of boosting trees using k-fold cross validation (10-fold cross variation in our case) and fits the model with the selected number of trees. The learning rate for each model was adjusted to achieve at least 1000 trees [35]. The tree complexity (the number of nodes in a tree) of 2 was used due to the relatively small sample size of 119 sites for each year, as increasing tree complexity to more than 2 does not improve predictive deviance when sample size is small [35]. The predictive performance of each model (*D*^2^) was based on the cross-validated proportion of the total deviance explained. We used a measure of relative influence of BRT explanatory variables based on the number of times a variable was selected for splitting and weighing each split by the model improvement. The relative influences of explanatory variables were scaled such that they totaled to 100 and presented as percentages. As we had a single set of environmental variables from GIS layers, we separately fitted BRT to data from 2020 and 2021 and focused on variables that had high relative influence in both 2020 and 2021 for interpretation.

## Results

### Abundance-occupancy relationship

**Fig 2:**
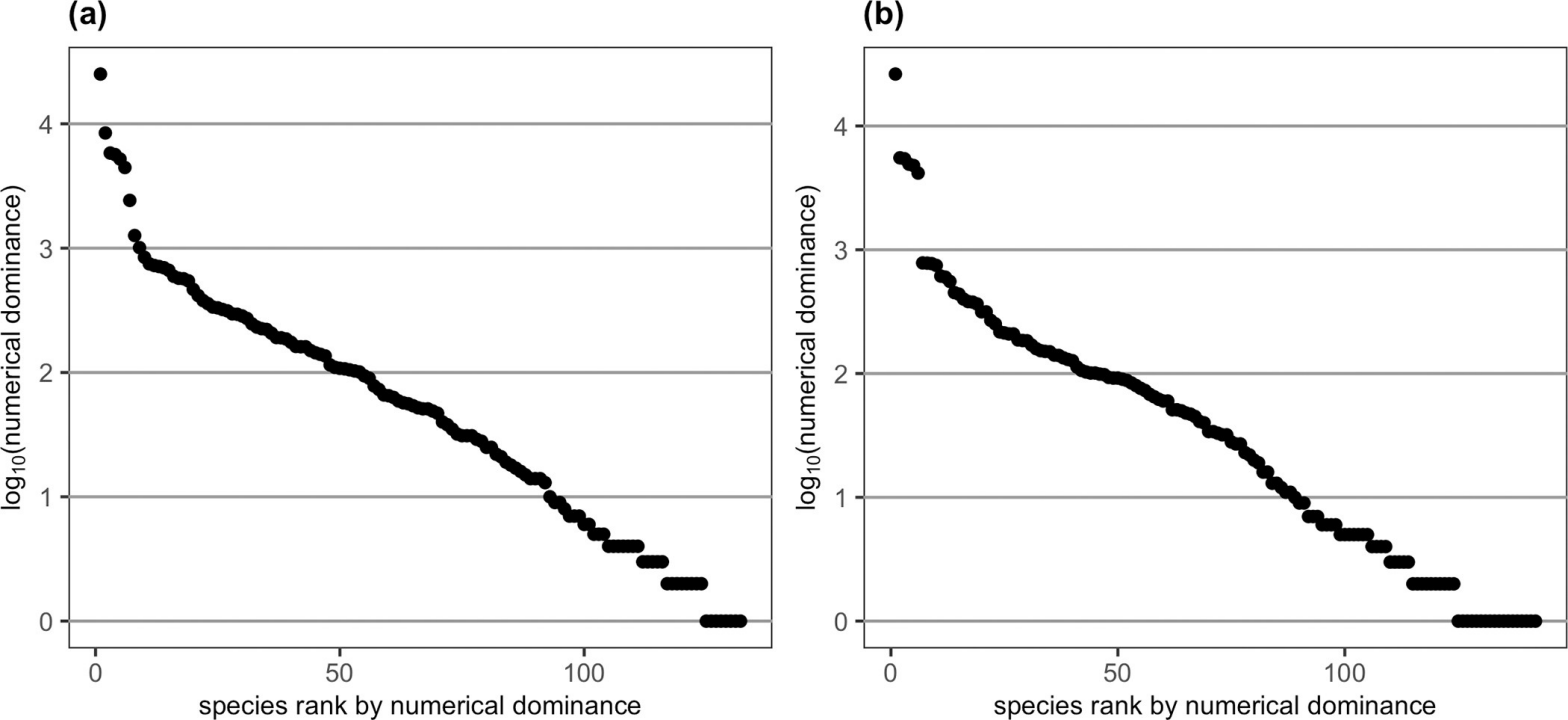
Numerical dominance of each species (log_10_) from the 119 survey sites, ranked by the numerical dominance for (a) 132 species in 2020 and (b) 142 species in 2021.

The total numbers of individuals and species from the 119 sites differed slightly between the two years, with 75,549 reef fish belonging to 132 species recorded in 2020 and 65,180 fish belonging to 142 species recorded in 2021. Numerical abundances of reef fish were dominated by a relatively small number of species, with the top 20 species (14–15% of all species) accounting for 88% and 90% of all individuals in 2020 and 2021, respectively. More than half of the species in the present study had the numerical dominance (total count from the 119 survey sites) of <119 (i.e., mean count of < 1 per site; Fig 2). For species occurrence, 32 species representing 90% of all individuals and 24 species also representing 90% of all individuals occurred at more than half of the survey sites in 2020 and 2021, respectively. The numerically dominant species (Table 2) generally corresponded with those species that occurred at more than half of the survey sites in either 2020 or 2021 (S1A and S1B Data).

**Table 2:** Top 20 reef fish species by numerical dominance in South Kona at 119 survey sites in 2020 and 2021.

2020	2021
*Chromis vanderbilti* (25,149) *Acanthurus nigrofuscus* (8,449) *Zebrasoma flavescens* (5,824) *Ctenochaetus strigosus* (5,659) *Chromis agilis* (5,216) *Thalassoma duperrey* (4,454) *Naso lituratus* (2,424) *Acanthurus leucopareius* (1,264) *Acanthurus olivaceus* (1,010) *Melichthys niger* (845) *Halichoeres ornatissimus* (748) *Parupeneus multifasciatus* (726) *Ctenochaetus hawaiiensis* (712) *Pseudocheilinus evanidus* (696) *Scarus psittacus* (664) *Chaetodon multicinctus* (594) *Plagiotremus goslinei* (573) *Sufflamen bursa* (568) *Plectroglyphidodon imparipennis* (546) *Acanthurus blochii* (466)	*Chromis vanderbilti* (26,280) *Acanthurus nigrofuscus* (5,533) *Chromis agilis* (5,436) *Zebrasoma flavescens* (4,914) *Ctenochaetus strigosus* (4,796) *Thalassoma duperrey* (4,167) *Pseudocheilinus evanidus* (783) *Plectroglyphidodon imparipennis* (780) *Naso lituratus* (773) *Halichoeres ornatissimus* (748) *Acanthurus olivaceus* (613) *Acanthurus leucopareius* (601) *Chaetodon multicinctus* (555) *Ctenochaetus hawaiiensis* (451) *Parupeneus multifasciatus* (438) *Plagiotremus goslinei* (400) *Canthigaster jactator* (382) *Sufflamen bursa* (378) *Pseudocheilinus octotaenia* (365) *Melichthys niger* (315) *Stethojulis balteata* (315)

The numerical dominance of each species is shown in the parentheses.

**Fig 3:**
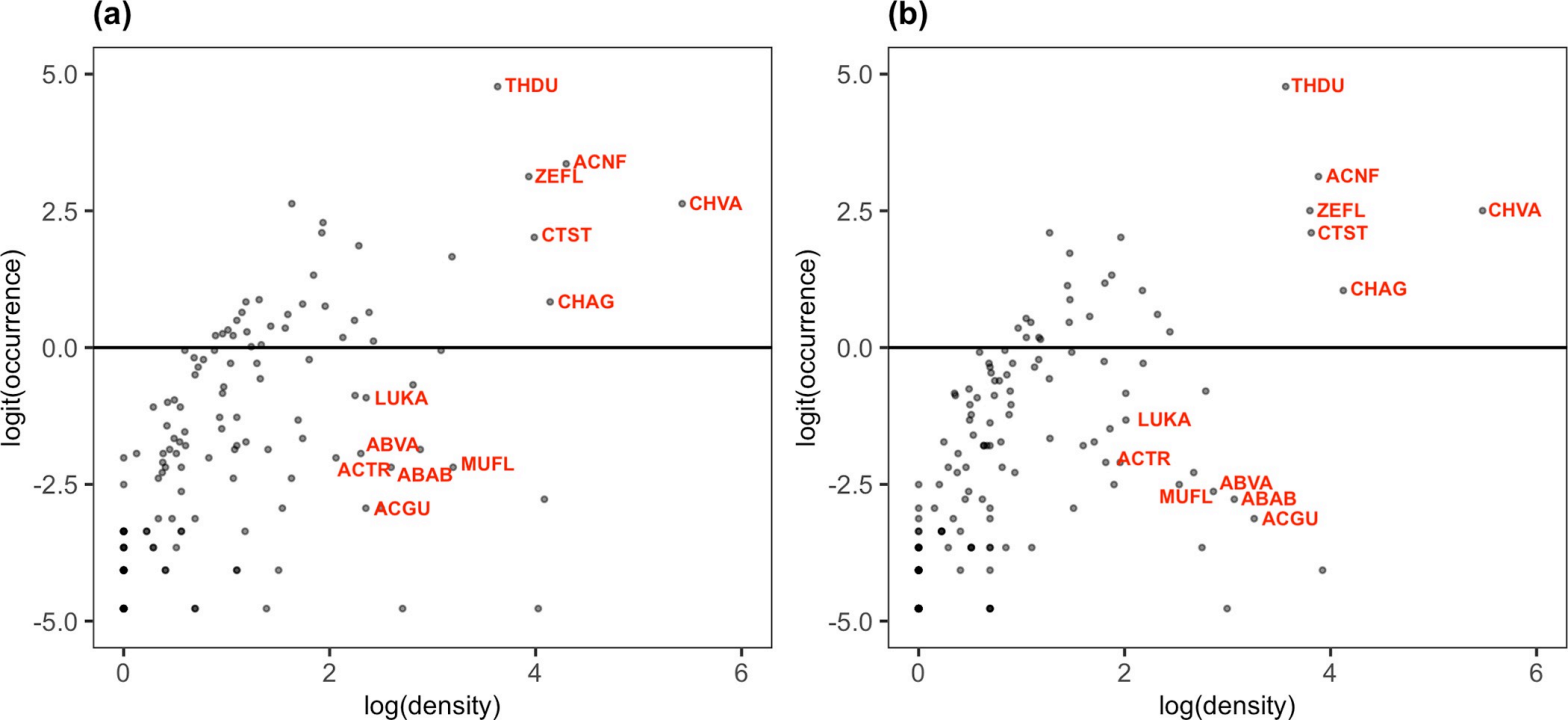
Plots of log-transformed mean density and logit-transformed occurrence (i.e. log[proportion of occurrence / proportion of non-occurrence]) for (a) 2020 and (b) 2021. The horizontal line at logit(occurrence) = 0 shows species occurring at more than half of the survey sites above the line and those occurring at less than half of the survey sites below the line. Fish species that consistently occurred widely in high abundance and ones that consistently appeared on the bottom right of the plots in both years are labeled in red by their species codes that combine the first two letters of the genus name and the first two letters of the species name.

Considering the mean density of each fish species (i.e., ignoring the sites with 0 counts for each species), plots of log-transformed mean density and logit-transformed occurrence (i.e., log[proportion of occurrence/proportion of non-occurrence]) showed statistically significant positive relationships in 2020 and 2021 (Fig 3). In these plots, species that occurred widely in high numerical abundance, such as *Chromis vanderbilti* (CHVA), *Acanthurus nigrofuscus* (ACNF), *Zebrasoma flavescens* (ZEFL), *Ctenochaetus strigosus* (CTST), *Chromis agilis* (CHAG) and *Thalassoma duperrey* (THDU), appear toward the top right, and moving toward the bottom left of each plot shows progressively rare species (Fig 3). Several species consistently appeared, however, on the bottom right of the plots in both years showing that they were not widespread but abundant within a site when they occurred. These species included *Abudefduf abdominalis* (ABAB), *Abudefduf vaigiensis* (ABVA), *Acanthurus guttatus* (ACGU), *Acanthurus triostegus* (ACTR), *Lutjanus kasmira* (LUKA) and *Mulloidichthys flavolineatus* (MUFL).

### Fish assemblage patterns

All environmental variables retained for multivariate DISTLM conditional tests (i.e., northing coordinates, depth, rugosity, housing density within 3-km radius of the nearest point on shore, the number of unique vegetation types within 3-km radius of the nearest point on shore, chlorophyll-*a* concentration, live coral cover, algal cover and sand cover) individually explained statistically significant proportions of variation in the fish assemblage structure on the basis of the Bray-Curtis dissimilarity in 2020 and 2021 (DISTLM marginal tests at α = 0.05). In 2020, water depth, sand cover and rugosity had the highest marginal correlations with the assemblage structure individually explaining 8.3%, 5.5% and 4.0% of the variation, respectively. Similarly, in 2021, water depth, rugosity and sand cover had the highest marginal correlations with the assemblage structure explaining 9.7%, 4.4% and 3.9% of the variation, respectively. The final parsimonious model based on AICc included the variables northing, depth, housing density, chlorophyll-*a* concentration, coral cover and sand cover for 2020 (DISTLM conditional test, *R*^2^ = 0.22; Table 3) and the variables northing, depth, housing density, chlorophyll-*a* concentration and sand cover for 2021 (DISTLM conditional test, *R*^2^ = 0.21; Table 4).

**Table 3:** Results of DISTLM conditional tests showing the final parsimonious model for the structure of 2020 fish assemblage as a whole on the basis of the Bray-Curtis dissimilarity after square-root transformation of fish count per species per site.

Variables	Explained SS	Pseudo-*F*	*P*	*R* ^2^
Northing	3042	2.46	0.0034	0.021
+ depth	12380	10.81	0.0002	0.104
+ housing density (3 km)	2755	2.45	0.0050	0.123
+ chlorophyll-*a*	3342	3.02	0.0008	0.145
+ coral cover	4690	4.37	0.0002	0.177
+ sand cover	5835	5.66	0.0002	0.217

**Table 4:** Results of DISTLM conditional tests showing the final parsimonious model for the structure of 2021 fish assemblage as a whole on the basis of the Bray-Curtis dissimilarity after square-root transformation of fish count per species per site.

Variables	Explained SS	Pseudo-*F*	*P*	*R* ^2^
Northing	5621	4.31	0.0002	0.036
+ depth	15231	12.85	0.0002	0.132
+ housing density (3km)	2865	2.45	0.0064	0.150
+ chlorophyll-*a*	3602	3.13	0.0008	0.173
+ sand cover	5676	5.12	0.0002	0.208

**Fig 4:**
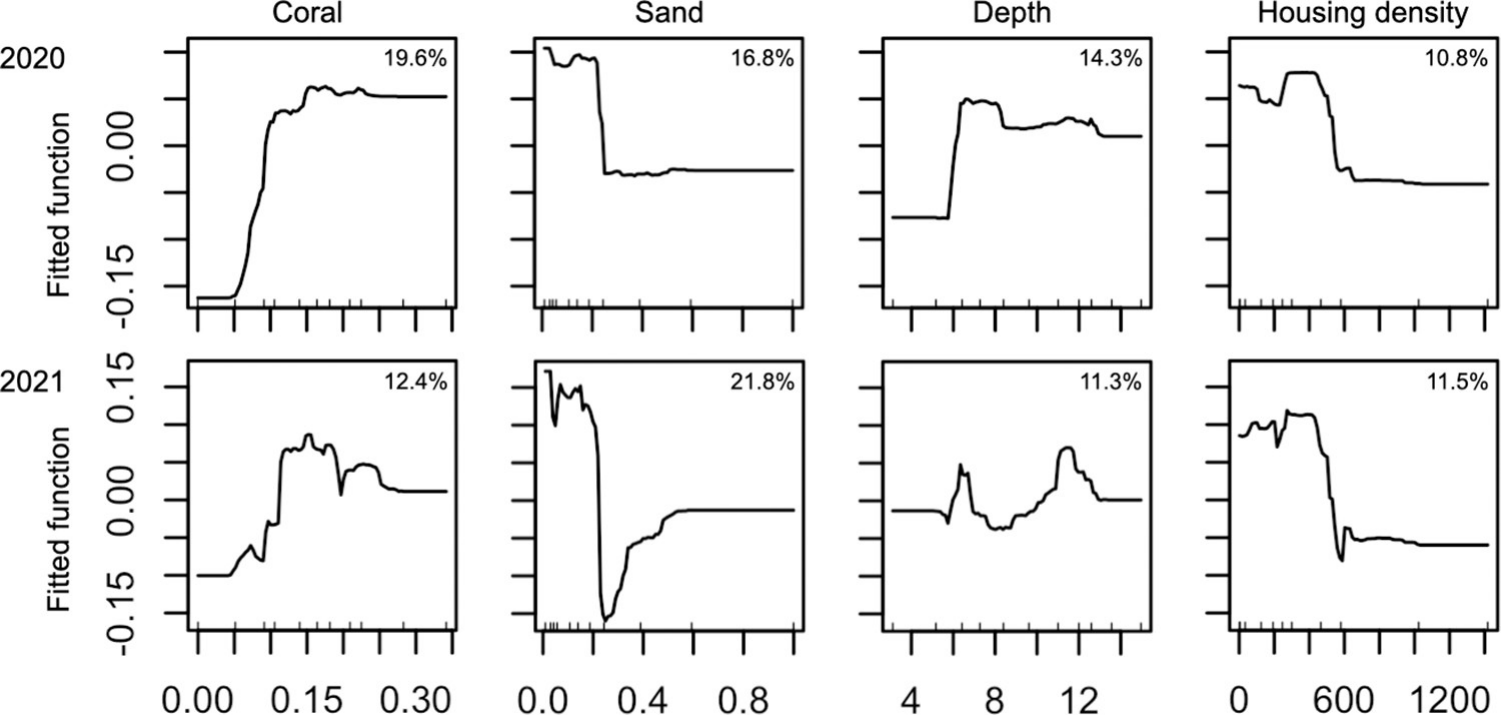
**Partial dependence plots for the environmental variables in the BRT models for the log-transformed total abundance that had relative influence of >10% in both 2020 and 2021.** The x axes show the environmental variables, proportion of coral cover, proportion of sand cover, depth (m) and housing density within 3-km radius of the nearest point on shore. The y axes show marginal effects of environmental variables on the log-transformed total fish abundance. The percentages in the plots show the relative influences of the environmental variables.

**Fig 5:**
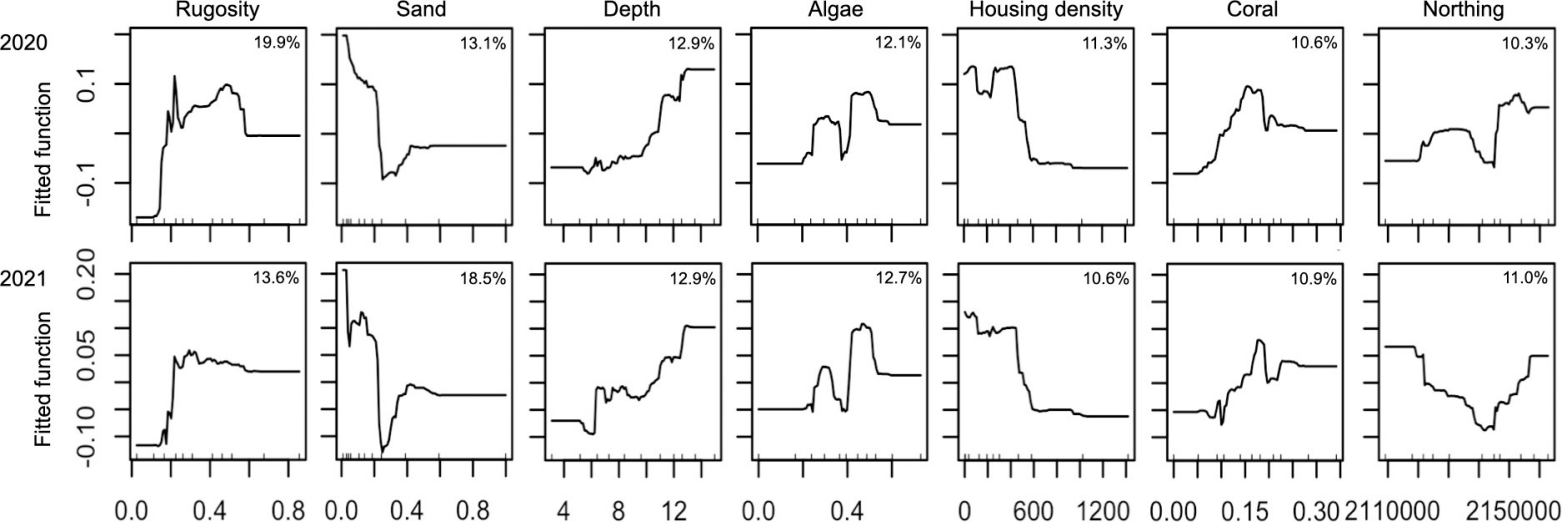
Partial dependence plots for the environmental variables in the BRT models for species richness that had relative influence of >10% in both 2020 and 2021. The x axes show the environmental variables, reefscape-level rugosity, proportion of sand cover, depth (m), proportion of algal cover, housing density within 3-km radius of the nearest point on shore, proportion of coral cover and northing coordinates. The y axes show marginal effects of environmental variables on fish species richness. The percentages in the plots show the relative influences of the environmental variables.

BRT models for univariate response variables had predictive performance (*D*^2^) ranging from 0.05 to 0.39, and *D*^2^ values varied across the two years except for species richness (Table 5). Total fish abundance was strongly influenced by live coral cover, sand cover, depth and housing density (S1 Table). The models predicted a high overall fish abundance for reefs with high coral cover and low sand cover at deeper depths with low housing density within 3-km radius of the nearest point on shore (Fig 4). Species richness was strongly influenced by rugosity, live coral, sand and algal cover, depth, housing density and northing coordinates (S1 Table). A high level of species richness was predicted for reefs with low sand cover and intermediate levels of live coral and algal cover and rugosity at deeper depths with low housing density (Fig 5). The influence of northing coordinates was somewhat inconsistent across the two years, but both models predicted the lowest species richness around the middle region of the surveyed coastline (Fig 5).

**Table 5:** Summary of BRT models showing the mean deviance for a null model (null deviance), mean residual deviance of the model (residual deviance), mean residual deviance based on 10-fold cross validation (CV deviance), cross-validated proportion of the total deviance explained (*D*^2^).

Variables	Year	Null deviance	Residual deviance	CV deviance	*D* ^2^
Total abundance	2020	0.075	0.033	0.066	0.123
	2021	0.079	0.009	0.048	0.389
Species richness	2020	2.994	0.639	2.308	0.229
	2021	2.686	0.594	2.076	0.227
Grazer	2020	0.160	0.056	0.135	0.156
	2021	0.165	0.113	0.150	0.094
Browser	2020	0.302	0.047	0.211	0.304
	2021	0.246	0.152	0.227	0.080
Scraper (P/A)	2020	1.005	0.761	0.913	0.092
	2021	1.288	1.090	1.246	0.033
Corallivore	2020	0.112	0.071	0.107	0.047
	2021	0.107	0.062	0.093	0.131
Resource fish	2020	0.191	0.070	0.164	0.140
	2021	0.125	0.095	0.119	0.046

CV deviance was based on 10-fold cross validation, dividing data into 10 subsets and using nine out of the 10 as training data, while withholding one as independent evaluation data to calculate residual deviance. *D*^2^ was based on the cross-validated proportion of the total deviance explained and shows the predictive performance of each model.

Influential explanatory variables varied among herbivorous trophic groups in the BRT analyses. Distributions of grazers and browsers were strongly influenced by rugosity and sand cover, as well as housing density for browsers (S1 Table). A high abundance of grazers was predicted for reefs with high rugosity and low sand cover (S1A Fig), while a high abundance of browsers was predicted for reefs with high rugosity, low sand cover and low housing density (S1B Fig). The presence of scrapers was strongly influenced by rugosity and live coral cover, with their presence being predicted on reefs with high rugosity and live coral cover (S1C Fig).

The distribution of corallivores was strongly influenced by live coral cover, sand cover and depth (S1 Table). A high abundance of corallivores was predicted for reefs with high coral cover and low sand cover at deeper depths (S1D Fig). The distribution of resource fish was strongly influenced by rugosity as well as sand and algal cover (S1 Table), with a high abundance being predicted for reefs with high rugosity and low sand cover, along with intermediate to high algal cover (S1E Fig).

## Discussion

The fish surveys in South Kona yielded mostly consistent results from 119 sites across the two years of the study. Overall, the fish assemblages were dominated by a relatively small number of species that widely occurred: a pattern of the previously documented positive abundance-occupancy relationship in ecology [36], including fish [37]. While fish abundance was dominated by the small-bodied planktivore, *Chromis vanderbilti*, the top 20 species by numerical dominance were represented by all trophic groups, including herbivores (grazers, browsers and scrapers), detritivores, corallivores, mobile and sessile invertivores, planktivores and piscivores (Table 2 and S1C Data). In addition to these widely occurring and abundant species, six species (*Abudefduf abdominalis*, *Abudefduf vaigiensis*, *Acanthurus guttatus*, *Acanthurus triostegus*, *Lutjanus kasmira* and *Mulloidichthys flavolineatus*) were consistently found in high abundance when they occurred. These six species are known to school or form feeding aggregations [38–40], explaining their deviations from the pattern of gradient between the widely occurring, abundant species and rare species (see Fig 3). Plotting the occurrence and mean density of each fish species as presented here allows for quick identification of potential schooling species in reef fish data and can offer valuable information in subsequent data analysis.

The structure of the fish assemblages in South Kona on the basis of the Bray-Curtis dissimilarity showed patterns consistent with those previously reported from other parts of Hawai‘i. The influence of depth on coral-reef fish assemblage structure has previously been shown from shallow to upper mesophotic depths [41], and the present study confirms the strong influence of depth in South Kona even in the narrow range of 3 to 15 m. When univariate variables were considered, species richness and corallivore abundance showed increases along the depth gradient (Fig 5 and S1D Fig), partially explaining the changes in the fish assemblage structure. For corallivore abundance, a previous study in the Northwestern Hawaiian Islands also showed their abundance to peak at 27 m [6]. Such changes in corallivore abundance with depth have important implication regarding the impact of coral bleaching on reef fish assemblages as increases in water temperature have disproportionate effects on corals, and in turn corallivores, along a depth gradient [42].

The influence of reef structural complexity on fish assemblages has been previously shown in Hawai‘i based on reef-level rugosity (e.g., rugosity based on a chain-and-tape method or the number of holes on a coral reef) [2, 5, 43]. The present study further demonstrated that reefscape-level rugosity based on a GIS layer at 2-m resolution can offer important information on the structure of reef fish assemblages. Despite the statistically significant marginal correlation in the DISTLM analyses, however, rugosity was not selected for the final parsimonious models in the DISTLM conditional tests (Tables 3 and 4), indicating a large overlap in the variation explained by this variable and one or more other environmental variables in the final models. For example, sand cover is the opposite of the total hard-bottom cover (i.e., non-sand cover, [the total reef area excluding sand (%) = 100%—sand cover (%)]). As sand cover consistently showed negative associations with fish abundance variables and species richness in the BRT analyses (Figs 4 and 5, S1B, S1D, S1E Fig), the pattern could be translated to positive correlations between the abundance of reef fish and the total hard-bottom cover, and thus reef structure.

Chlorophyll-*a* concentration had strong correlations with the structure of fish assemblages on the basis of the Bray-Curtis measure, but it was not identified as an influential variable in any of the univariate BRT analyses (S1 Table). These results have an important implication as they indicate species-specific associations between chlorophyll-*a* concentration and fish abundance; counteracting species-specific changes in abundance (i.e., some positive and others negative) can result in no statistically significant effects when the data were lumped into the univariate metrics disregarding the identity of individual species. In a previous study across the Pacific islands, chlorophyll-*a* concentration, which was used as an indicator of oceanic productivity, had overall positive effects on reef fish biomass [44], but the spatial scale of our study is much smaller. As chlorophyll-*a* concentration was highly correlated with average sea surface temperature and wave height in the present study (see the Methods for details), this variable could reflect local physical conditions of the reefs in South Kona. Physical environmental variables such as water motion, wave exposure and sea surface temperature can affect the distribution of reef fishes [4, 23, 43].

While herbivores (grazers, browsers and scrapers) in the present study consistently showed their tendency to occur on reefs with a high level of reefscape-level structural complexity, group-specific associations with environmental variables were also found. Browsers were the only group that exhibited a negative correlation with housing density (S1B Fig), showing their tendency to occur in higher abundance away from human populations. This could be due to the vulnerability of browsers to fishing pressure, as browsing surgeonfishes are highly desired targets in Hawai‘i and across the Pacific [23]. On the other hand, the presence of scrapers was positively correlated with live coral cover. On the island of O‘ahu, the abundance of scarids (mostly *Chlorurus spilurus*, *Scarus psittacus* and *Scarus rubroviolaceus*) was also found to be positively correlated with live coral cover [45]. While some levels of corallivory have been reported among large scarids [46, 47], live coral accounts for a very small proportion of their diet in Hawai‘i [48]. Scrapers can facilitate recruitment and growth of coral by closely cropping reef substratum and increasing the space for settlement [49, 50]. Therefore, the association detected between the presence of scrapers and live coral cover could be due to scrapers enhancing live coral cover, rather than scrapers responding to the distribution of live coral as preferred habitat or food sources.

Unlike the abundance of corallivores that was strongly influenced by live coral cover (i.e., their food source), the distributions of herbivores were not affected by algal cover (S1 Table). These patterns are consistent with a previous study in the Northwestern Hawaiian Islands [5]. Among the three types of herbivores in the present study, browsers feed on macroalgae whereas grazers and scrapers feed on epilithic turf algae [50, 51]. As the GIS layer for algal cover in the present study primarily captures macroalgae, there is no surprise that the abundance of grazers and presence of scrapers were not affected by the algal cover variable in the present study. It also should be noted that the negative relationship found between sand cover and grazer abundance (S1A Fig) could be due to positive responses of grazers to the total hard-bottom cover and the availability of turf algae. For browsers, algal cover was not identified as influential due to its relative influence of <10% in 2021. However, the relative influence in 2021 was 9.7%, close to 10% (S1 Table), and the models for both years predicted high algal cover to result in a higher abundance of browsers. As the predictive performance of the model for browsers in 2021 was relatively low (Table 5), further studies are needed to determine whether macroalgal cover as a food source affects browser abundance in Hawai‘i.

The abundance of resource fish was strongly influenced by rugosity, sand cover and algal cover. In a previous study in West Hawai‘i, Foo et al. (2021) [9] found a positive correlation between rugosity and the biomass of adult resource fish. Our study further confirmed this association for the abundance of resource fish regardless of their size. The resource fish species in the present study consisted of nine grazers, four browsers, four scrapers, 12 invertivores, two planktivores, one corallivore and four piscivores (S1C Data). As the resource fish in the present study primarily consisted of herbivores and invertivores, the patterns detected by the analyses likely reflected the distributions of these fishes, explaining the similarity between the results of the analyses for herbivores and resource fish. In addition, macroalgae host a variety of epifaunal invertebrates [52], and increases in certain species of invertivores have been found with an increase in macroalgal cover [53]. In the same study, the abundance of browsers also slightly increased with increasing algal cover [53]. Investigating the distribution of individual species is beyond the scope of this study because individual species abundances were too sparse to model their individual distributions, but future studies targeting specific species, particularly invertivores and browsers, likely offer further insights into the effects of algal cover on the distributions of reef fishes.

This study intensively surveyed shallow coral reefs along the coastline of South Kona over two years and was the most complete spatial survey on the reef fish assemblages to date. The patterns found in the present study were overall consistent with previous studies conducted either at larger spatial scales across the Pacific or in other parts of Hawai‘i, indicating the influences of various environmental factors including depth, total hard-bottom cover, reefscape-level rugosity, human population density and live coral cover on the fish assemblages. The effects of environmental variables specific to trophic groups were also found, highlighting the importance of considering their traits when examining the relationships between fish and habitat. While BRT are sometimes criticized for the tendency to overfit data reducing model generality (i.e., the differences between residual deviance and CV deviance in Table 5), their flexibility allows for an accurate description of the relationship in the data [35]. Nevertheless, variations in the data explained by the model based on residual deviance, as well as predictive performance based on CV deviance (i.e., *D*^2^), were relatively low for some of the BRT models, indicating the presence of other factors affecting the fish assemblages. As we utilized GIS layers to examine large-scale patterns in the fish assemblages, further studies should include in-situ environmental data for more detailed investigations. Such investigations incorporating potential local factors including, for example, fine-scale structural complexity created by coral and macroalgae as habitat engineers, can reveal further insights into the relationships between the habitat and fish assemblages in Hawai‘i.

## Supporting information

S1 Table Results of univariate BRT models. Explanatory variables with relative influence of >10% for both 2020 and 2021 models are shown in bold.
(XLSX)

S1 Fig Partial dependence plots for BRT models. The plots show the models for [a] the log-transformed grazer abundance, [b] the log-transformed browser abundance, [c] the presence/absence of scrapers, [d] the log-transformed corallivore abundance and [e] the log-transformed resource fish abundance.
(PDF)

S1 Data Summary data of all species recorded in 2020 and 2021. The data show [a] numerical dominance, occurrence and mean density of each species in 2020, [b] numerical dominance, occurrence and mean density of each species in 2021, and [c] species codes, trophic categories and whether each species was considered resource fish.
(XLSX)

S1 File Raw data. Fish and environmental variables used in the present study.
(ZIP)
