# Supplementary material for: Fish assemblage structure, diversity and controls on reefs of South Kona, Hawaiʻi Island
Source: PLoS One. 2023 Jul 6;18(7):e0287790. doi: 10.1371/journal.pone.0287790 (PMC10325036; doi:10.1371/journal.pone.0287790)

S1 Fig [a]. Partial dependence plots for the environmental variables in the models for the log-transformed grazer abundance that had relative influence of >10% in both 2020 and 2021. The x axes show the environmental variables, reefscape-level rugosity and proportion of sand cover. The y axes show marginal effects of environmental variables on the log-transformed grazer abundance. The percentages in the plots show the relative influences of the environmental variables.

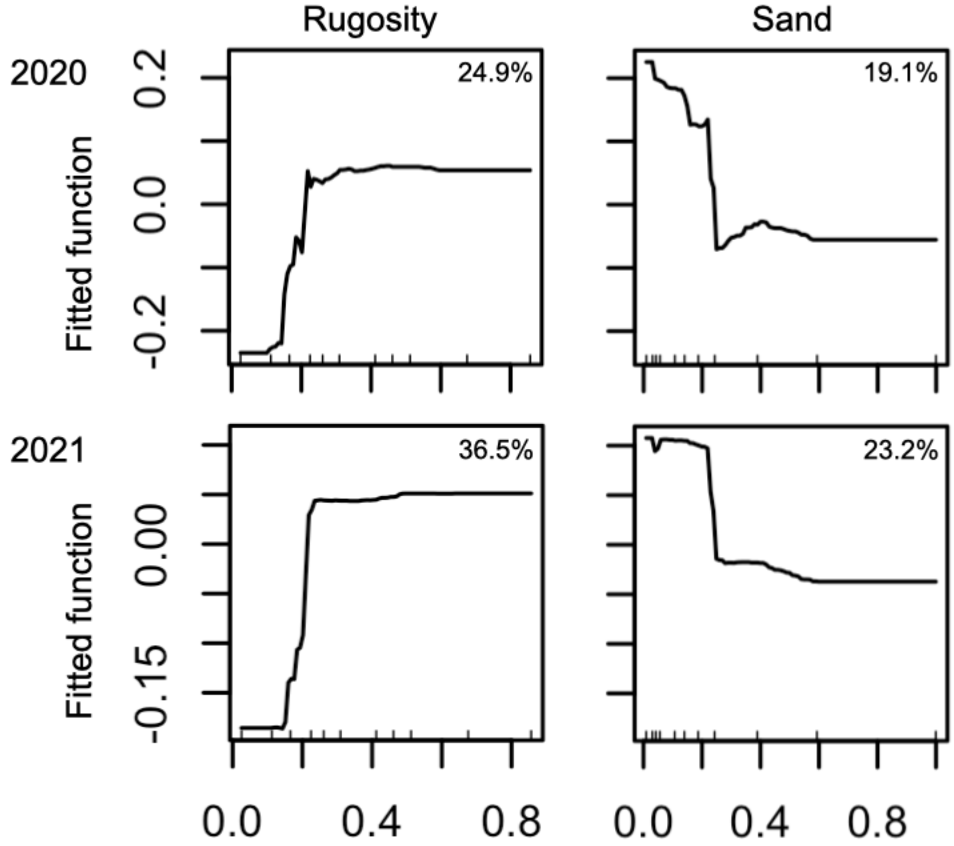

S1 Fig [b]. Partial dependence plots for the environmental variables in the models for the log-transformed browser abundance that had relative influence of >10% in both 2020 and 2021. The x axes show the environmental variables, proportion of sand cover, housing density within 3-km radius of the nearest point on shore and reefscape-level rugosity. The y axes show marginal effects of environmental variables on the log-transformed browser abundance. The percentages in the plots show the relative influences of the environmental variables.

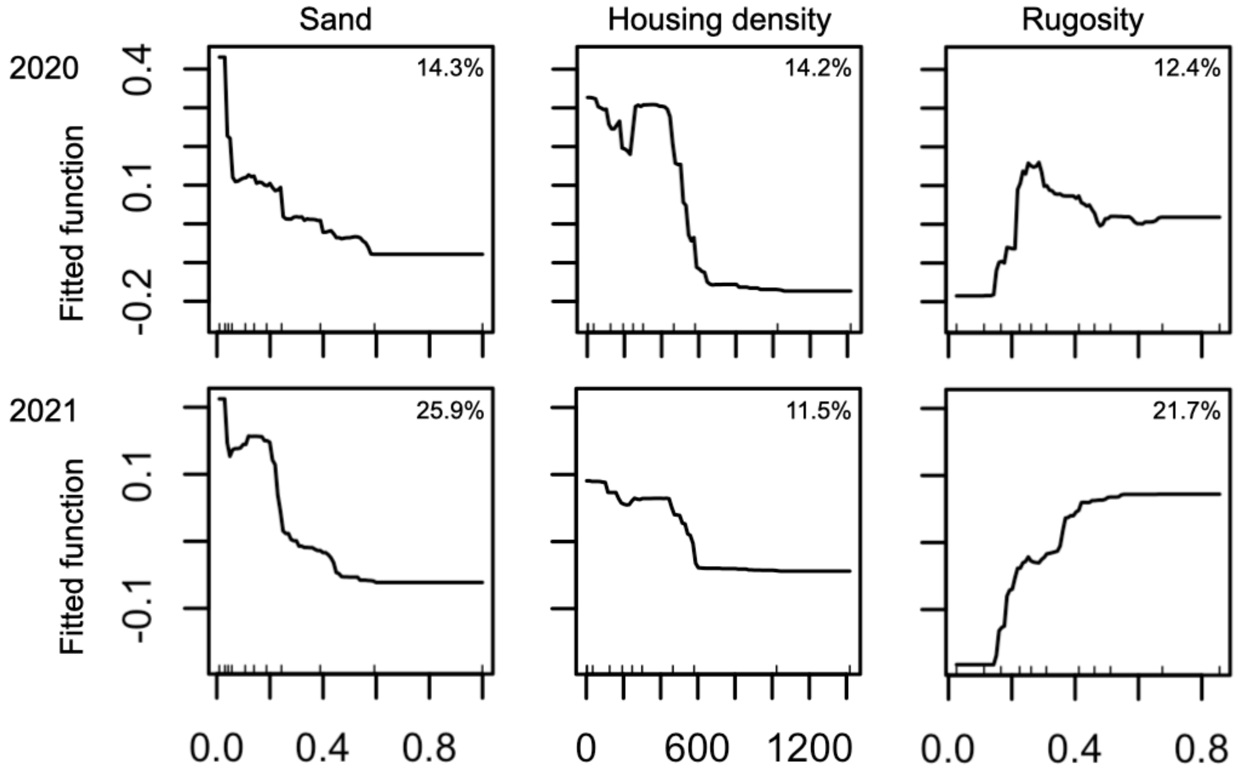

S1 Fig [c]. Partial dependence plots for the environmental variables in the models for the presence/absence of scrapers that had relative influence of >10% in both 2020 and 2021. The x axes show the environmental variables, reefscape-level rugosity and proportion of coral cover. The y axes show marginal effects of environmental variables on the presence/absence of scrapers. The percentages in the plots show the relative influences of the environmental variables.

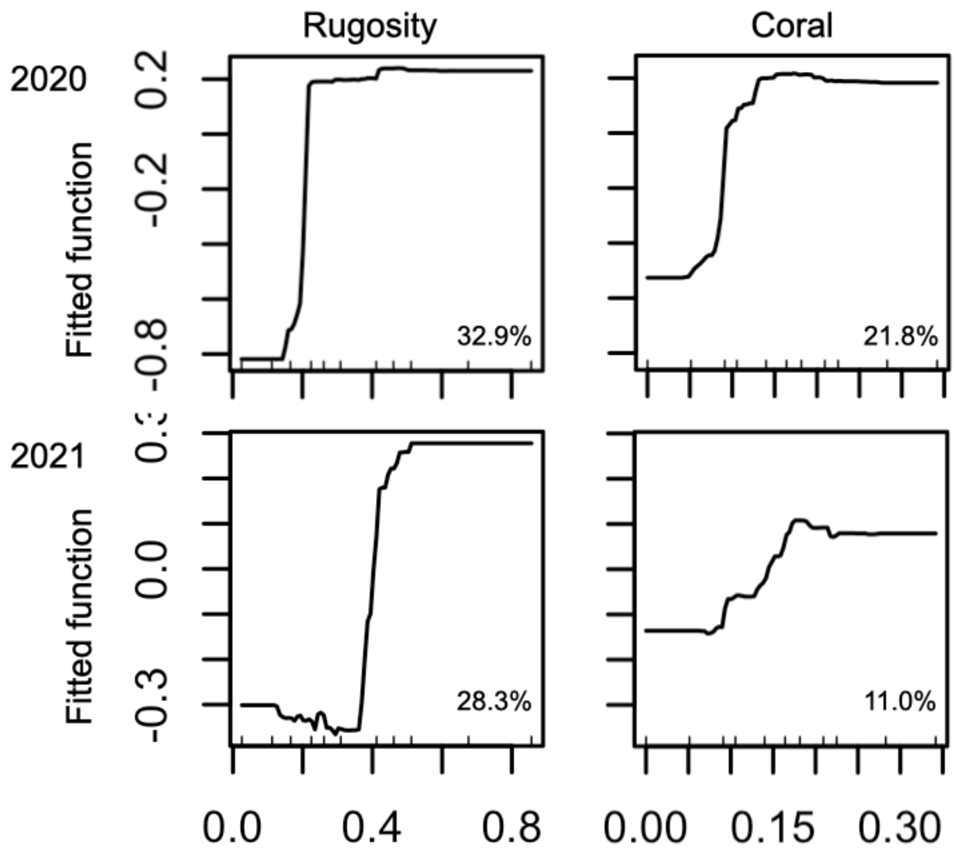

S1 Fig [d]. Partial dependence plots for the environmental variables in the models for the log-transformed corallivore abundance that had relative influence of >10% in both 2020 and 2021. The x axes show the environmental variables, proportion of coral cover, depth (m) and proportion of sand cover. The y axes show marginal effects of environmental variables on the log-transformed corallivore abundance. The percentages in the plots show the relative influences of the environmental variables.

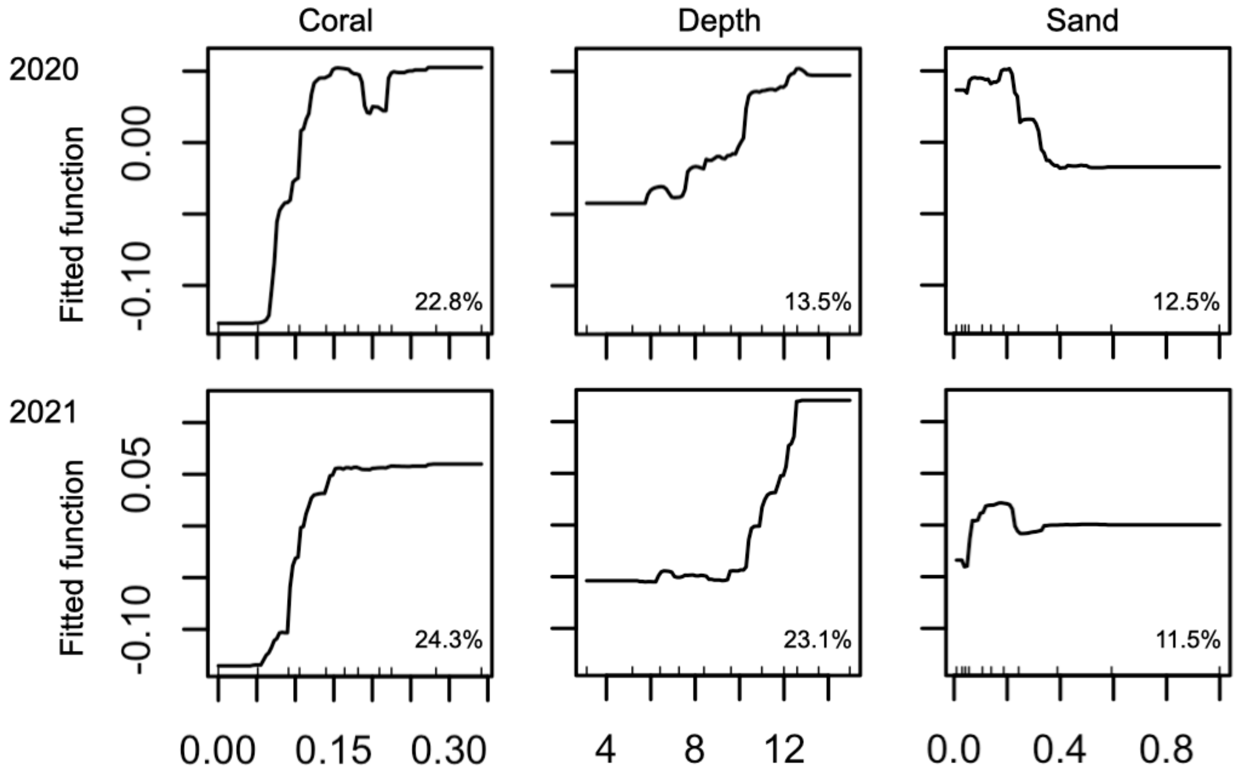

S1 Fig [e]. Partial dependence plots for the environmental variables in the models for the log-transformed resource fish abundance that had relative influence of >10% in both 2020 and 2021. The x axes show the environmental variables, reefscape-level rugosity, proportion of sand cover and proportion of algal cover. The y axes show marginal effects of environmental variables on the log-transformed resource fish abundance. The percentages in the plots show the relative influences of the environmental variables.

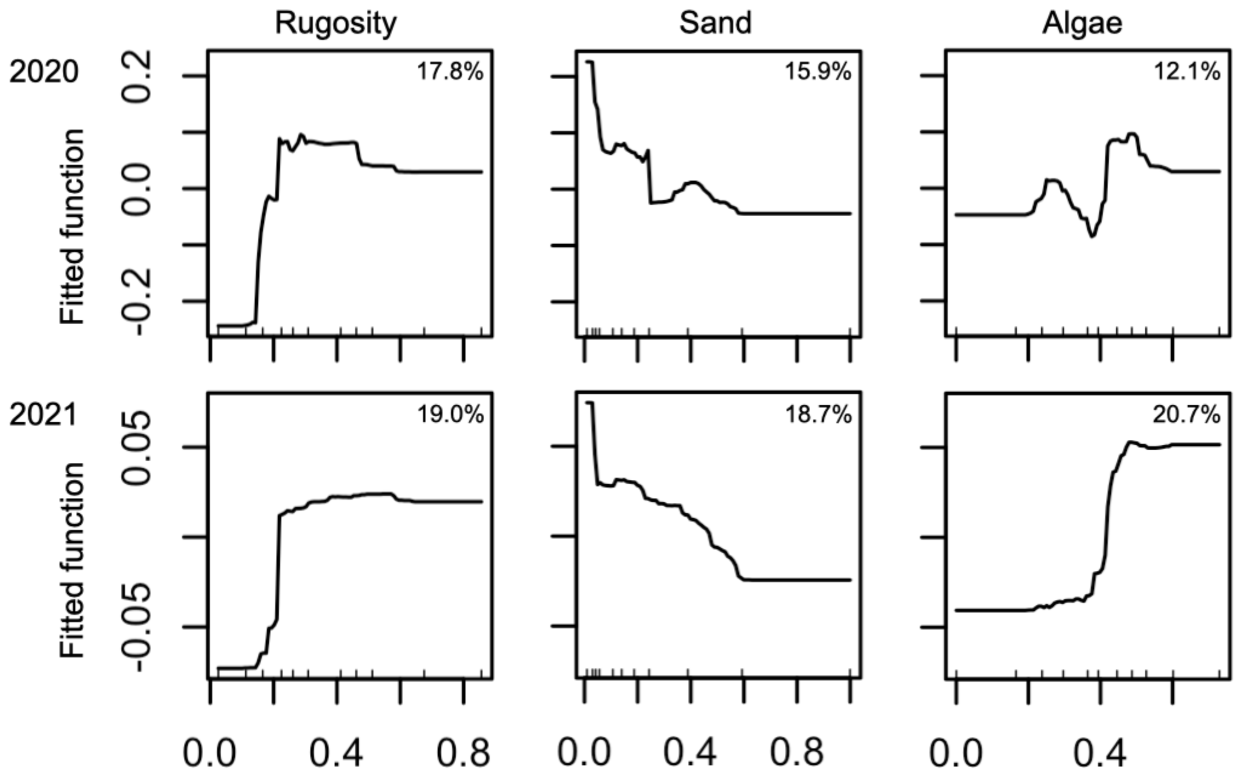

Supplement: S1 Fig — The plots show the models for [a] the log-transformed grazer abundance, [b] the log-transformed browser abundance, [c] the presence/absence of scrapers, [d] the log-transformed corallivore abundance and [e] the log-transformed resource fish abundance. (PDF) [file pone.0287790.s002.pdf]
